# Low arterial oxygen partial pressure induces pulmonary thrombocytopenia in patients and a mouse model

**DOI:** 10.1186/s12890-020-01381-7

**Published:** 2021-01-06

**Authors:** Limeng Wu, Ninghong Guo, Zhenyan Xu, Wei Wang, Qinmei Xiong, Jinzhu Hu, Rong Wan, Kui Hong

**Affiliations:** 1 Department of Cardiovascular Medicine and Jiangxi Key Laboratory of Molecular Medicine, The Second Affiliated Hospital of Nanchang University, Nanchang, 330006 Jiangxi China; 2 Department of Hematology Medicine, The Second Affiliated Hospital of Nanchang University, Nanchang, China; 3 Molecular Medicine of Jiangxi Key Laboratory, The Second Affiliated Hospital of Nanchang University, Nanchang, China; 4 Department of Respiratory Medicine, The Second Affiliated Hospital of Nanchang University, Nanchang, China; 5 Department of Genetic Medicine, The Second Affiliated Hospital of Nanchang University, Nanchang, China

**Keywords:** Pulmonary infection, Thrombocytopenia, Lung, Platelet, Oxygen partial pressure

## Abstract

**Background:**

Recent basic studies demonstrate that the lung is a primary organ of platelet biogenesis. However, whether the pathophysiological state of the lung affect the platelets is little known. We aim to investigate the incidence of thrombocytopenia in patients with pulmonary infection (PIN) and risk factors associated with pulmonary thrombocytopenia.

**Methods:**

In total, 11,941 patients with pulmonary infection (PIN) were enrolled, and patients with other three infectious diseases were collected as controls. The incidence of thrombocytopenia was compared, and the risk factors associated with thrombocytopenia in PIN patients were investigated by multivariate analysis. To explore the mechanism of thrombocytopenia, hypoxic model was constructed. Blood platelet counts from the angular vein (PLTs), left ventricle (PLT_post_) and right ventricle (PLT_pre_) were determined. Megakaryocytes identified by anti-CD41 antibody were detected through flow cytometry and immunofluorescence.

**Results:**

The incidence of thrombocytopenia in PIN was higher than that in other three infectious diseases (9.8% vs. 6.4% ~ 5.0%, *P* < 0.001). Low arterial oxygen partial pressure (PaO_2_) was an important risk factor for thrombocytopenia (OR = 0.88; *P* < 0.001). In a hypoxic mouse model, PLTs decreased (518.38 ± 127.92 vs 840.75 ± 77.30, *P* < 0.05), which showed that low PaO_2_ induced thrombocytopenia. The difference between the PLT_post_ and PLT_pre_ (∆PLT_post-pre_), representing the production of platelets in the lungs, was significantly attenuated in hypoxic mice when compared with normoxic mice (F = 25.47, *P* < 0.05). Additionally, proportions of CD41-positive megakaryocytes in the lungs, marrow, spleen all decreased in hypoxic mice.

**Conclusion:**

There is a high incidence for thrombocytopenia in PIN patients. Low PaO_2_-induced thrombocytopenia is associated with impaired generation of platelet in the lungs.

## Background

Platelets are critical for hemostasis and thrombosis [1]. It is a classic view that megakaryocytes (MKs) produce platelets in the bone marrow. Interestingly, several studies have shown that a large mass of MKs exist in lungs, which indicates that the lungs may be a specific organ for platelet biogenesis [2–4]. The latest discovery strongly indicated that a large number of MKs circulated through the lungs, where they dynamically released platelets, and an animal model showed that the lungs contributed approximately 50% of total platelet production in mice [3]. It is often observed that thrombocytopenia appears in patients with pulmonary diseases, such as pneumonia, chronic obstructive pulmonary disease (COPD) or respiratory failure (RF), which leads to major bleeding events and death [4–7]. However, whether pathophysiological state of lung affects platelets is unclear from both clinical and basic research information. The present study, firstly, aims to explore whether the risk of thrombocytopenia varies among different organ infection, and we found that the incidence of thrombocytopenia in pulmonary infection (PIN) was the highest and low oxygen partial pressure (PaO_2_) was a key risk factor for thrombocytopenia in PIN. Secondly, mice hypoxia model were used to uncover the potential mechanism of thrombocytopenia associated with the lung.

## Methods

### Subjects

Data were obtained from the large data center of the Second Affiliated Hospital of Nanchang University from January 1, 2014, to June 30, 2018. International classification of diseases code-10 (ICD-10) was applied to identify the diagnosis of PIN [8]. Additionally, patients with urinary tract infection (UTI), intestinal tract infection (ITI) or skin soft-tissue infection (SSI) were collected as controls to distinguish PIN-specific effects of infection. All subjects meeting any of the following criteria were excluded: (1) younger than 18 years old; (2) pregnant or lactating; (3) presence of infection at two or more sites; (4) a total bilirubin, alanine aminotransferase or aspartate aminotransferase level 1.5 times higher than the normal value or a serum creatinine level 1.2 times higher than the normal value [9]; (5) presence of any disorder that could lead to thrombocytopenia, including hematopoietic disease, cancer, hepatitis, cirrhosis, hypersplenism, autoimmune disease, disseminated intravascular coagulation and hemorrhagic fever; and (6) concomitant intake of medication that could affect the platelet, including chemotherapeutic drugs, monoclonal antibodies, antiplatelet drugs, anticoagulant drugs, and some antibiotics (e.g. linezolid and vancomycin). The clinical pulmonary infection score (CPIS), which is used to assess the severity of PIN [10] and the Acute Physiology And Chronic Health Evaluation II (APACHE II) score, which is used to estimate the severity of RF [11], were calculated with data collected by a third person blinded to the experimental design to avoid bias. Thrombocytopenia was defined as a platelet count lower than 100 × 10^9^/L, which was defined according to the corresponding statement by the World Health Organization [12]. All procedures were approved by the Medical Ethics Committee of the Second Affiliated Hospital of Nanchang University.

### Mouse models

Sixteen C57BL/6 male mice (20–28 g, age 10 weeks) were purchased from Laboratory Animal Science Department of Nanchang University. To verify whether the PaO_2_ is associated with thrombocytopenia, we generated a respiratory failure model in mouse and the protocol was well-accepted and utilized by other researchers [13]. The mice were randomly assigned into two groups. Hypoxic mice (N = 8) were housed in a hypobaric chamber with an 8% O_2_ concentration and 58–66% humidity for 28 continuous days, while control mice (N = 8) were housed in normal air conditions. The mice were kept under conditions with controlled lighting (12 h per day) and temperature (21 ± 2 °C) and were free to standard laboratory food and water. Four mice were placed in one cage. At the end of 28-day hypoxia treatment, mice were anesthetized by inhaling 5% isoflurane and blood was obtained quickly, then all mice were euthanized by cervical dislocation and the tissues were collected. In order to ensure the repeatability, experiment were performed on independent days as separate replicates. All procedures were approved by the Animal Care and Use Committee of the Second Affiliated Hospital of Nanchang University.

### Platelet counts and P-selectin assessment

Mice were anesthetized by inhalation of isoflurane(RWD, China) before blood collection. The platelet counts of blood samples collected from different sites including the angular vein (PLTs), the right ventricle (PLT_pre_) and the left ventricle (PLT_post_) were determined with an automatic blood cell analyzer(Bio-rad TC20, USA). Plasma soluble P-selectin, a marker of platelet activation, was evaluated by enzyme-linked immunosorbent assay (Invitrogen, USA).

### Flow cytometry

To obtain a single-cell suspension, 100 mg lung was digested with LiberaseTM (Roche, Germany)at a concentration of 26 U/ml and 5% DNase I in a 37 °C warm bath for 30 min, followed by filtration through a 100-μm sieve; and cell suspensions of bone marrow and spleen were performed as described previously [14, 15]. Cell suspensions treated with 1 ml RBC lysis buffer (Solarbio, China) were incubated with anti-CD41 FITC-conjugated antibody (eBioscience, USA) in the dark for 30 min and then evaluated by flow cytometry [16]. IgG FITC-conjugated isotype control antibody(eBioscience, USA) was applied to gated.

### Tissue immunofluorescence

Lung, femur and spleen of mice were rapidly removed after euthanasia and fixed with 4% paraformaldehyde for 24 h. Fixed tissue specimens were dehydrated in ethanol, infiltrated, embedded into paraffin. For an individual mouse, 5 sections were randomly cut along the direction of the maximum section of the tissue. Sections (2-mm thick) were incubated with anti-CD41 primary antibody (Proteintech, USA) in PBS containing 1% BSA overnight at 4 °C [17], followed by secondary antibody conjugated to Alexa Fluor 488 or 568 (Invitrogen Corporation, Molecular Probes) for 1 h at room temperature. Finally, the slide was observed by a fluorescence microscope(OLYMPUS, JAPAN) and analyzed by the software(Image-Pro Plus 6.0., CHINA). The number of CD41-positive cells per square millimeter was calculated.

### Statistical analysis

Statistical analysis was performed by using SPSS 13.0 software (SPSS Inc. Chicago, Illinois, USA). Continuous variables are expressed as the mean ± standard deviation (*x* ± *SD*), while categorical variables are presented as relative frequencies. Logistic regression models were used to identify clinical factors associated with thrombocytopenia. All covariates that reached statistical significance (*P* < 0.05) in univariate analyses were selected for multivariate analysis. An independent *t* test was used for comparisons between two groups if the data conformed to a normal distribution. Otherwise, the Mann-Whitney U test was used. A paired *t*-test was used to compare the PLT_pre_ and the PLT_post_. To compare the difference in the ∆PLT_post-pre_ value between two groups, a covariance analysis model was used. A *P* value < 0.05 was considered statistically significant.

## Results

### Patient cohort

**Fig. 1:**
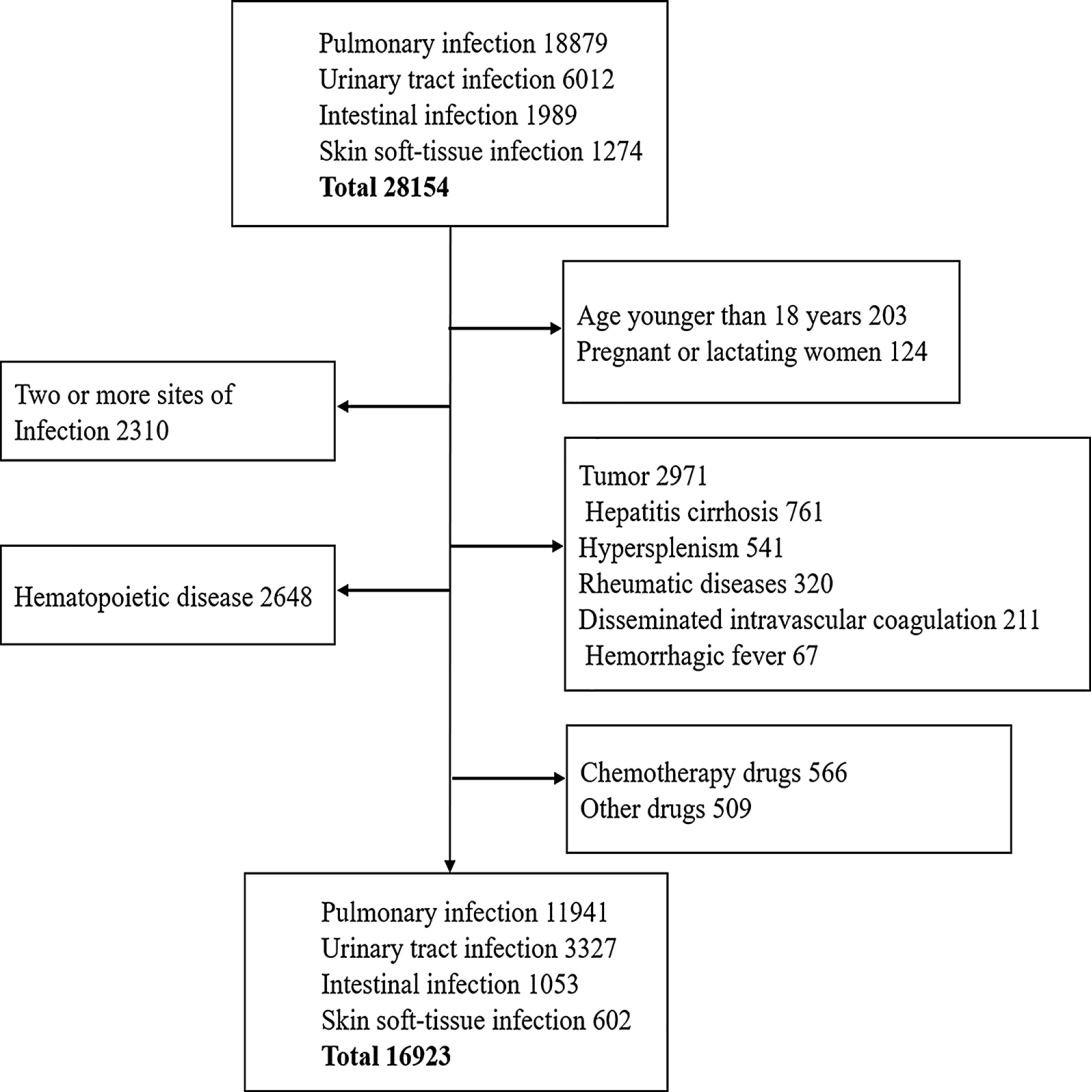
Flowchart showing the process of this study

A total of 11,941 (70.5%) patients with PIN were enrolled, while 3327(19.7%) patients with UTI, 1053 (6.2%) patients with ITI, and 602 (3.6%) patients with SSI were collected to be used as controls. The initial cohort included 16,923 patients (Fig. 1), and the baseline characteristics are presented in Table 1. The incidence of thrombocytopenia were 9.8% (PIN), 6.4% (UTI), 5.0% (ITI) and 5.1% (SSI).

**Table 1:** Baseline Characteristics of Patients with Infectious Diseases (N = 16,923)

Variables	Thrombocytopenia (n = 1468)	Normal platelet (n = 15,455)	*p* Value
*Demographic characteristics*			
Age, years	57.94 ± 23.62	58.57 ± 23.81	0.34
Sex, female, n (%)	876(59.7)	8771(56.8)	0.03*
Infection location, n (%)			< 0.001*
Lung	1171(9.8)	10,770(90.2)	
Urinary tract	213(6.4)	3114(93.6)	
Intestinal tract	53(5.0)	1000(95.0)	
Skin soft-tissue	31(5.1)	571(94.9)	
*Biochemical indexes*			
WBC (*10^9^/L)	9.92 ± 3.99	10.00 ± 4.09	0.54
HB (g/L)	128.46 ± 33.44	127.45 ± 33.42	0.27
PLT (*10^9^/L)	70.33 ± 23.53	211.32 ± 80.26	< 0.001*
TBil (μmol/L)	19.94 ± 8.62	19.81 ± 8.53	0.58
AST (U/L)	36.07 ± 13.60	36.49 ± 13.66	0.26
ALT (U/L)	42.17 ± 18.80	41.84 ± 19.08	0.54
Creatinine (mmol/l)	93.86 ± 30.21	93.30 ± 30.18	0.49
CRP (mg/L)	50.48 ± 48.53	51.21 ± 49.21	0.67
PCT (ng/ml)	0.35 ± 0.21	0.25 ± 0.35	0.60
*Medical history, n (%)*			
Hypertension	453 (30.9)	5512 (35.7)	< 0.001*
Diabetes	220 (15.0)	2613 (16.9)	0.06

Date were presented as mean ± (SD) or n(%).The physiological variables were collected within the first 24 h of admission. ^*^Significant differences (*P* < 0.05)

*WBC* white blood cell, *HB* haemoglobin, *PLT* platelet, *TBil* total bilirubin, *AST* aspertate aminotransferase, *ALT* alanine aminotransferase, *CRP* C-reactive protein, *PCT* procalcitonin

### Patients with PIN showed a higher incidence of thrombocytopenia than patients with other infectious diseases

For all subjects with infectious disease, factors associated with thrombocytopenia by logistic analysis are shown in Table 2. By univariate analysis, sex, hypertension comorbidity and different infection sites were associated with thrombocytopenia. In multivariate analysis, after adjusting for the sex and hypertension comorbidity confounders, patients with other organ infections presented a lower incidence of thrombocytopenia than PIN patients did (UTI OR = 0.61, 95% CI: 0.52–0.71; ITI OR = 0.48, 95% CI: 0.33–0.69; SSI OR = 0.46, 95% CI: 0.35–0.61; all *P* < 0.001), which indicates that patients with PIN are more likely to develop thrombocytopenia than patients with one of three other infections.

**Table 2:** Comparison of the incidence of thrombocytopenia between patients with pulmonary infection and other infections

Variables	Univariate analysis			Multivariate analysis		
	OR	95% CI	*P* value	OR	95% CI	*P* value
Age, years	0.99	0.99–1.00	0.33			
Sex, females	1.13	1.01–1.26	0.03^*^	1.09	0.98–1.22	0.14
Infection Sites						
Lung	1			1		
Urinary tract	0.63	0.54–0.73	< 0.001*	0.61	0.52–0.71	< 0.001^*^
Intestinal tract	0.49	0.37–0.65	< 0.001^*^	0.48	0.33–0.69	< 0.001^*^
Skin soft-tissue	0.50	0.35–0.72	< 0.001^*^	0.46	0.35–0.61	< 0.001^*^
Biochemical indexes						
WBC(× 10^9^/L)	0.99	0.90–1.09	0.54			
HB(g/L)	1.00	0.99–1.03	0.27			
CRP(mg/L)	0.99	0.99–1.03	0.59			
PCT(ng/ml)	1.11	0.09–1.15	0.60			
Medical history, n (%)						
Hypertension	0.81	0.72–0.90	< 0.001^*^	0.74	0.66–0.84	< 0.001^*^
Diabetes	0.87	0.75–1.01	0.06			

*WBC* white blood cell, *HB* haemoglobin, *CRP* C-reactive protein, *PCT* procalcitonin, *OR* odds ratio, *CI* confidence interval

*Significant differences (*P* < 0.05)

### PIN patients with RF comorbidity had a relatively high risk for thrombocytopenia

To explore the risk factors for thrombocytopenia in PIN patients, factors influencing thrombocytopenia were identified by logistic analysis (Table 3). The CPIS was evaluated to assess the severity of PIN [10]. Univariate analysis showed that the higher CPIS was (OR = 1.25, 95% CI: 1.18–1.32; *P* < 0.001), the higher the risk of thrombocytopenia. RF (OR = 1.68, 95% CI: 1.35–2.09; *P* < 0.001) and hypertension (OR = 0.73, 95% CI: 0.65–0.83; *P* < 0.001) were associated with thrombocytopenia. Multivariate analysis showed that hypertension was a positive factor for thrombocytopenia (OR = 0.74, 95% CI: 0.65–0.85; *P* < 0.001). RF (OR = 1.59, 95% CI: 1.27–1.98; *P* < 0·001) and a high CPIS (OR = 1.24, 95% CI: 1.17–1.31; *P* < 0.001) were risk factors for thrombocytopenia. Interestingly, patients with RF appeared more prone to thrombocytopenia than those without RF.

**Table 3:** Analysis on Risk Factors Associated with Thrombocytopenia in Patients with Pulmonary Infection (N = 11,941)

Variables	Thrombocytopenia (n = 1171)	Normal platelet (n = 10,770)	Univariate Analysis			Multivariate Analysis		
			OR	95%CI	*P *value	OR	95% CI	*P* value
Age, years (SD)	57.79 ± 24.06	58.76 ± 23.74	0.99	0.99–1.00	0.19			
Sex, female, n (%)	454 (38.8)	4356 (40.4)	0.93	0.82–1.06	0.27			
*Biochemical indexes*								
WBC (× 10^9^/L)	9.90 ± 3.98	9.96 ± 4.08	0.99	0.98–1.01	0.63			
HB (g/L)	126.75 ± 33.31	127.81 ± 33.49	0.98	0.98–0.99	0.30			
CRP (mg/L)	49.54 ± 48.18	51.23 ± 49.29	0.99	0.98–1.00	0.26			
PCT (ng/ml)	0.25 ± 0.15	0.31 ± 0.10	0.98	0.97–1.99	0.87			
CPIS scores	7.25 ± 2.16	7.00 ± 2.99	1.25	1.18–1.32	< 0.001^*^	1.24	1.17–1.31	< 0.001^*^
*Medical history, n (%)*								
Hypertension	392 (33.5)	4385 (40.7)	0.73	0.65–0.83	< 0.001^*^	0.74	0.65–0.85	< 0.001^*^
Diabetes	177 (15.1)	1798 (16.7)	0.89	0.75–1.05	0.17			
RF	101 (8.6)	574 (5.3)	1.68	1.35–2.09	< 0.001^*^	1.59	1.27–1.98	< 0.001^*^
COPD	78 (6.7)	519 (4.8)	1.24	0.91–1.68	0.17			

Date were presented as mean ± (SD) or n(%)

*WBC* white blood cell, *HB* haemoglobin, *CRP* C-reactive protein, *PCT* procalcitonin, *CPIS* clinical lung infection score, *RF* respiratory failure, *COPD* chronic obstructive lung disease, *OR* odds ratio, *CI* confidence interval

*Significant differences (*P* < 0.05)

### Low PaO_2_ was a key risk factor for thrombocytopenia

To explore the key effective factor in PIN patients with RF, a subgroup analysis was conducted and the result is shown in Table 4. The APACHE II scoring system was adopted here to estimate the severity of RF [11]. Univariate analysis showed that the higher the APACHE II score was (OR = 1.09, 95% CI: 1.02–1.15; *P* = 0.007), the higher the risk of thrombocytopenia was. Relatively low PaO_2_ (OR = 0.88, 95% CI: 0.85–0.91; *P* < 0.001), hypertension (OR = 0.64, 95% CI: 0.41–0.99; *P* = 0.046) and COPD (OR = 2.35, 95% CI: 1.22–4.53; *P* = 0.01) were associated with thrombocytopenia. In multivariate analysis, both COPD (OR = 2.40, 95% CI: 1.22–4.76; *P* = 0.01) and a high APACHE II score (OR = 1.06, 95% CI: 1.01–1.13; *P* = 0.03) were risk factors for thrombocytopenia. It was important to emphasize that low PaO_2_ was a potential risk factor for thrombocytopenia, which was supported by result of relatively high PaO_2_ associated with a relatively low risk of thrombocytopenia (OR = 0.88, 95% CI: 0.85–0.92; *P* < 0.001). There was no difference in the distribution of PaO2 among different pathogen groups (*P* = 0.11) (Additional file 1: Fig. 1). Likewise, pathogens weren’t associated with thrombocytopenia in PIN patients with RF (all *P* > 0.05) (Additional file 2: Table 1).

**Table 4:** Analysis on risk factors associated with thrombocytopenia in pulmonary infection patients accompanying by respiratory failure (N = 675)

Variables	Thrombocytopenia (n = 101)	Normal platelet (n = 574)	Univariate analysis			Multivariate analysis		
			OR	95% CI	*P* value	OR	95% CI	*P* value
Age, years (SD)	58.92 ± 25.00	58.25 ± 23.45	1·00	0·99–1·01	0·79			
Sex, female, n (%)	34 (33.7)	170 (29.6)	1·21	0·77–1·89	0·41			
*Biochemical indexes*								
WBC (× 10^9^/L)	9.48 ± 3.84	9.94 ± 4.01	0.97	0.92–1.02	0.28			
HB (g/L)	126.39 ± 33.65	129.61 ± 33.32	0.09	0.99–1.01	0.37			
CRP (mg/L)	45.39 ± 44.97	49.94 ± 48.43	0.99	0.99–1.00	0.38			
PCT (ng/ml)	1.5 ± 1.2	1.6 ± 1.1	0.99	0.99–1.01	0.56			
APACHEII scores	13.3 ± 3.9	12.3 ± 3.5	1.09	1.02–1.15	0.007*	1.06	1.01–1.13	0.03*
*ABGA*								
PH	7.39 ± 0.2	7.38 ± 0.2	1.08	0.30–3.90	0.90			
PaO_2_ (mmHg)	46.2 ± 7.5	52.8 ± 5.8	0.88	0.85–0.91	< 0.001*	0.88	0.85–0.92	< 0.001*
PaCO_2_ (mmHg)	39.5 ± 16.1	38.7 ± 16.8	1.00	0.99–1.02	0.64			
*Medical history, n (%)*								
Hypertension	34 (33.7)	255 (44.4)	0.64	0.41–0.99	0.046*	0.65	0.41–1.05	0.08
Diabetes	16 (15.8)	120 (21.0)	0.71	0.40–1.26	0.24			
COPD	34 (33.7)	128 (22.3)	2.35	1.22–4.53	0.01*	2.40	1.22–4.76	0.01^*^

Date were presented as mean ± (SD) or n(%)

*WBC* white blood cell, *HB* haemoglobin,* CRP* C-reactive protein, *PCT* procalcitonin, *APACHE II scores* Acute Physiology and Chronic Health Evaluation, *ABGA* arterial blood gas analysis, *PaO*_*2*_ oxygen partial pressure, *PaCO*_*2*_ partial pressure of carbon dioxide, *COPD* chronic obstructive lung disease, *OR* odds ratio, *CI* confidence interval

*Significant differences (*P* < 0.05)

### Hypoxic mouse models with low PaO_2_

To verify whether low PaO_2_ is a risk factor for thrombocytopenia, hypoxic mouse models were constructed [13] to analyze the blood gas content in left ventricular blood. The results showed that the PaO_2_ (mmHg) decreased significantly in blood from hypoxic mice (n = 8) compared with that from normoxic mice (59.63 ± 6.39 *vs.* 76.63 ± 9.58, respectively; *P* < 0.05). For each Comparation, the sample size was eight.

### Reduction in PLTs independent of platelet activation after hypoxia

The count of PLTs (× 10^9^/L) in angular vein blood decreased in hypoxic mice compared with that of normoxic mice (518.38 ± 127.92 *vs.* 840.75 ± 77.30, respectively; *P* < 0.05) and accompanied by increased hemoglobin (HGB) level (g/L) (196.0 ± 10.56 vs. 140.0 ± 5.78, respectively; *P* < 0.05). There was no difference in the plasma soluble P-selectin concentration(ng/ml) between these two groups (77.55 ± 5.38 *vs.* 74.86 ± 7.85, respectively; *P* > 0.05). For each comparation, the sample size was eight.

### MK numbers decreased in hematopoietic organs after hypoxia

**Fig. 2:**
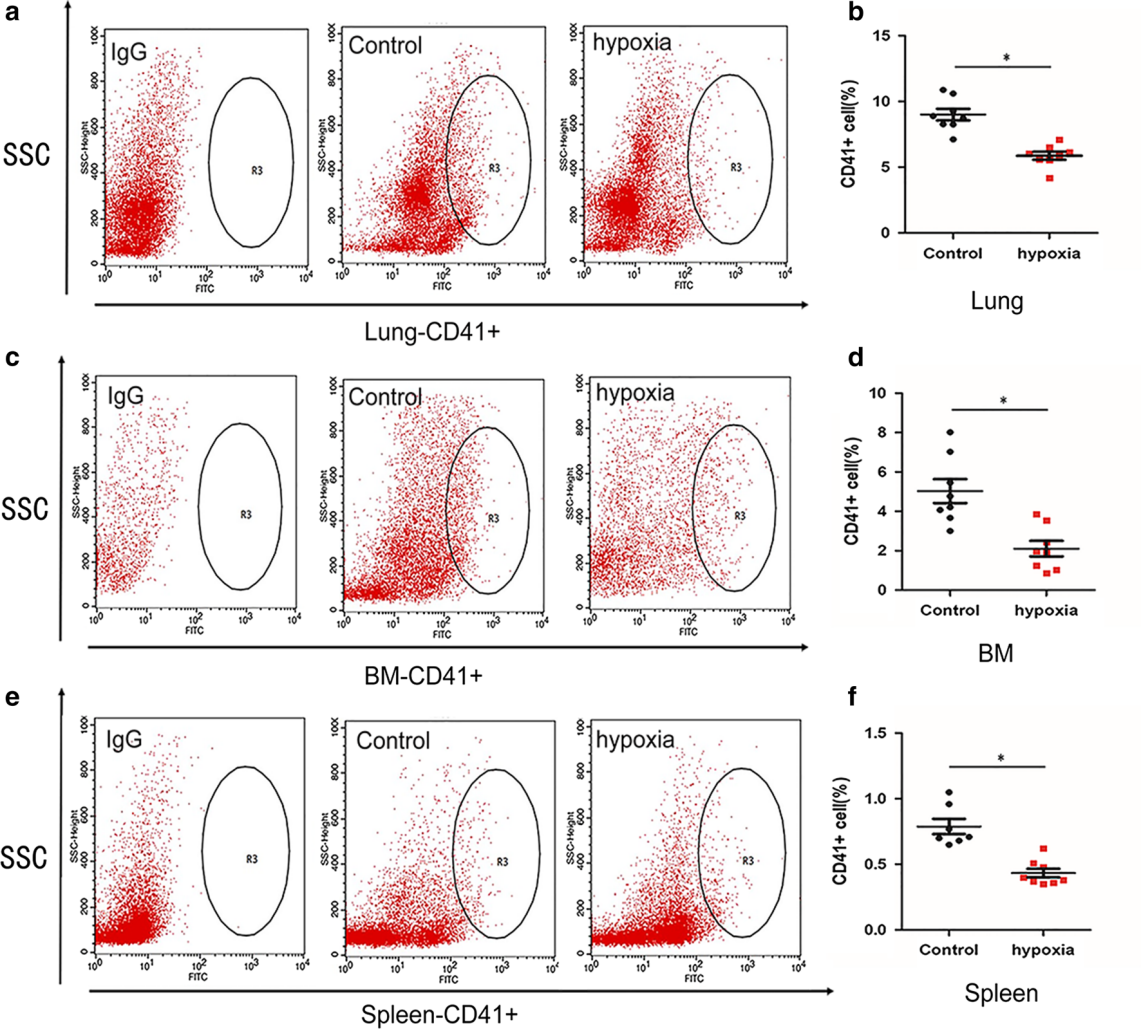
Hypoxia reduce the megakaryocytes in lung and other hematopoietic tissues (flow cytometry). Area for R3 define the amount of positive cells (determined bythe IgG-control isotypes). **a** Representative dot-plots of the staining of cell suspensions from the lung; **b** Comparation of the proportion of megakaryocytes in lungs between the two groups. Each point represents the mean adjusted value of 3 replicates for each individual mouse(n = 8). *P* values were calculated using the Mann–Whitney U test. *P < 0.05; **c** Representative dot-plots of the staining of cell suspensions from the bone marrow; **d** Comparation of the proportion of megakaryocytes in the BW between the two groups. Each point represents the mean adjusted value of 3 replicates for each individual mouse(n = 8). *P* values were calculated using the t test. *P < 0.05; **e** Representative dot-plots of the staining of cell suspensions from the spleen; **f** Comparation of the proportion of megakaryocytes in the spleen between the two groups (n = 8). *P* values were calculated using the Mann–Whitney U test. *P < 0.05

**Fig. 3:**
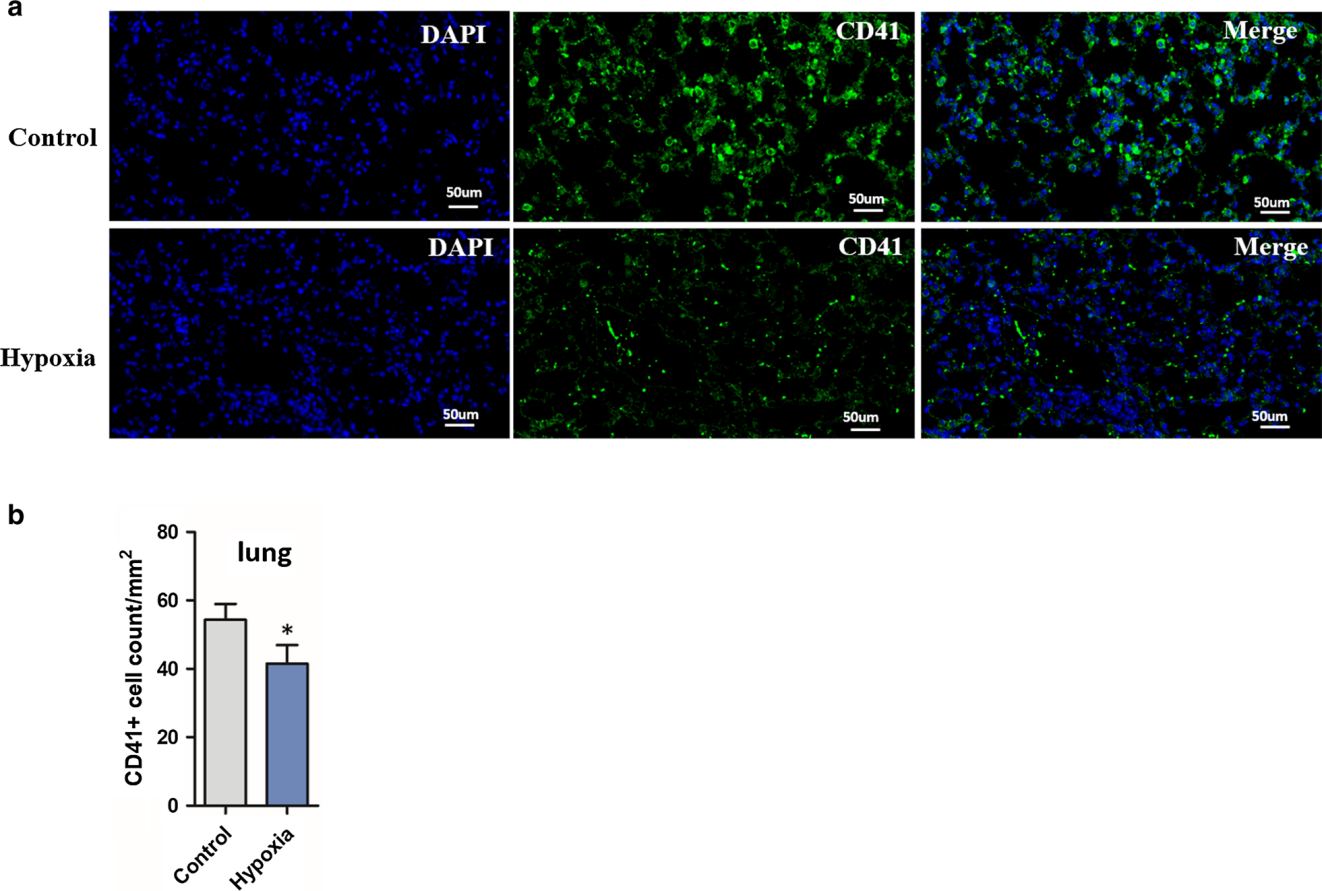
Hypoxia reduce the megakaryocytes in lung(immunofluorescence). **a** In representative images from sections of the lung, MKs (green, CD41) and nuclei (blue, DAPI) were showed. Scale: 50um; **b** Comparation of the megakaryocyte number between the two group. The y axis shows the number of CD41-positive cells per square millimeter. The data in the graphs are the means ± s.e.m. (n = 8), *p* values were calculated with the two-tailed Student t-test. *P < 0.05

To explore the possible reason for thrombocytopenia after hypoxia treatment, we investigated the amount of megakaryocytes in hematopoietic organs including lung, marrow and spleen. The results of flow cytometry analysis showed lower proportions of CD41-positive cells (%) in lungs (6.09 [5.66–6.71] *vs.* 8.82 [8.26–10.27], respectively; *P* < 0.05, Fig. 2a,b), bone marrow (2.11 ± 1.12 *vs.* 5.03 ± 1.72, respectively; *P* < 0.05, Fig. 2c,d) and spleen (0.39 [0.36–0.59] *vs.* 0.74 [0.66–1.03], respectively; *P* < 0.05, Fig. 2e–f) in hypoxic mice than in normoxic mice. Consistent with the flow cytometry results, the counts of CD41-positive cells calculated by fluorescence microscopy in lungs (39.0 ± 5.35 vs. 54.25 ± 12.87, respectively; *P* < 0.05, Fig. 3a,b), bone marrow (10.00 ± 2.78 vs. 24.88 ± 3.68, respectively; *P* < 0.05, Additional file 3: Fig. 2A–B) and spleen (2.75 ± 1.04 vs. 8.75 ± 5.29, respectively; *P* < 0.05, Additional file 4: Fig. 3A–B) in hypoxic mice were lower than those in normoxic mice. These results suggested that the reduction of megakaryocytes in the three hematopoietic organs after hypoxia might be part of the reason for thrombocytopenia. We speculate a perturbed PLT production in lung with the effect of hypoxia.

### Impaired platelet production was observed in hypoxic mouse lungs

To determine the number of platelets produced in lungs, we investigated the PLT_pre_ and the PLT_post_. The result showed thatthe PLT_post_ was higher than the PLT_pre_ in the normoxic group (713.63 ± 124.15 *vs.* 543.75 ± 121.17, respectively; *P* < 0.05), which indicated that thrombopoiesis occured in lungs. However, this phenomenon was not obvious in hypoxic mice (339.63 ± 95.47 *vs.* 391.13 ± 117.30, respectively; *P* > 0.05). Namely, the △PLT_post-pre_ value was less significant in the hypoxic group than in the normoxic group (F = 25.47, *P* < 0.05). For each Comparation, the sample size was eight.

## Discussion

There have been some cases of thrombocytopenia in PIN patients, but observational study of large samples is rare. We performed a retrospective study to observe the incidence of thrombocytopenia in patients with PIN, and patients with one of three other kinds of infections which was the most common in our hospital were chosen as controls. The results showed that the highest incidence of thrombocytopenia occurred in PIN patients among the four groups of infectious disease patients, suggesting that thrombocytopenia was likely associated with pulmonary infection. Subgroup analysis showed that PIN patients with RF had a higher risk of thrombocytopenia than those without RF. Furthermore, PIN with RF patients showing low PaO_2_ were more likely to have thrombocytopenia. These results indicated that low PaO_2_ might be a key risk factor for thrombocytopenia. In view of studies showing that the lungs can produce platelets [18, 19], it was reasonable to speculate that low PaO_2_ might induce thrombocytopenia by impairing platelet production in lungs. To verify our hypothesis, we built a hypoxic mouse model, and the results showed that PLTs were decreased in hypoxic mice compared with normoxic mice, which demonstrated that low PaO_2_ indeed induced thrombocytopenia. In keeping with the fact that MKs circulate through the pulmonary capillaries where they release platelets[19], the PLT_post_ representing the postpulmonary(left ventricle) blood platelet, was increased compared with the PLT_pre_ indicating the prepulmonary(right ventricle) blood platelet in normoxic mice. Hence, the △PLT_post-pre_ index represented the generation of platelets in lungs [20, 21]. Our results showed that △PLT_post-pre_ was significantly attenuated in hypoxic mice compared with normoxic mice. The lower proportion of CD41-positive MKs indicated by histology and flow cytometry, and the decreased △PLT_post-pre_ in hypoxic mice confirmed the speculation that low PaO_2_ could reduce MKs and impair the thrombocytopoiesis in lungs.

Although infection is known to cause thrombocytopenia [22–24], cohort studies associated with different organ infections have not been reported. In the present study, the incidence of thrombocytopenia in PIN patients showed a significant increase, which suggested that the lungs could affect the physiological behavior of platelets in a particular way. Based on the conclusion that there were no associations between bacterial species and the incidence of thrombocytopenia in infectious diseases [23], we speculate that lower PaO_2_ might cause pulmonary thrombocytopenia.

The correlation between low PaO_2_ and thrombocytopenia had been previously described. A clinical observation showed that thrombocytopenia occurred in 31% of neonates with asphyxia versus 5% of matched controls without asphyxia [25]. Another study found that thrombocytopenia was a predictive factor for the progression of pneumonia to RF [26]. We confirmed in clinical cases that low PaO_2_ was a key risk factor for thrombocytopenia through a relatively large sample of PIN patients for the first time. Severity of disease was associated with the incidence of thrombocytopenia [22, 24]. There was a relatively high incidence of thrombocytopenia ranging from 20 to 50% in critical patients [27]. Both the CPIS and the APACHE II score were positively associated with the risk of thrombocytopenia. It was worth mentioning that low PaO_2_ was an independent risk factor for thrombocytopenia after adjusting for the APACHE II score, which makes the results more convincing.

The influence of hypoxia on bone marrow MKs is well described. Chronic hypoxia impair bone marrow MKs [28] and inhibit the differentiation of bone marrow MKs[29], or the erythroid system and the MK system share a common precursor in the bone marrow, and there is competition between erythroid and MK differentiation upon exposure to a hypoxic environment [30]. However, the effect of hypoxia on pulmonary thrombocytopoiesis has not been investigated as the lung is another important site of platelet biogenesis. We constructed hypoxic mouse models and found that low PaO_2_ caused thrombocytopenia. P-selectin, an indicator of platelet activation, showed no significant difference between hypoxic and normoxic mice, which indicated that thrombocytopenia was not attributed to platelet activation. Researchers have paid close attention to the process of platelet generation in lungs [3, 19]. There are abundant MKs in the pulmonary arterial blood but only a few MKs in the pulmonary venous blood under normal conditions [21], but thrombocytopenia occurs in patients with congenital heart diseases because a right-to-left shunt bypasses the lung where thrombocytopoiesis occurs [31]. A large number of MKs dynamically circulate through the lungs, where they release platelets [19]. Consistent with these findings, we observed a large number of CD41-positive MKs in the mouse lungs and found that the PLT_post_ was higher than the PLT_pre_, indicating that the mouse lungs indeed were a site of platelet production. Interestingly, we found that hypoxia could reduce lung MKs and impair efficacy of thrombocytopoiesis in lung.

The conclusion from the present study could be helpful for patients with respiratory diseases. We discovered an interesting relationship between thrombocytopenia and pulmonary infections, as well as the corresponding hypoxemia. We found that megakaryocytes decreased in lung of hypoxia mice who produce only few platelets, suggesting that hypoxemia could result in reduced platelets and increased the risk of bleeding. Hypoxemia was common in patients suffering from COPD or bronchiectasis, or living in high altitude, so the clinicians **s**hould be aware of the risk of pulmonary thrombocytopenia**.** According to these reasons**,** we suggest that patients with severe lung diseases, especially those with hypoxemia complications should dynamically monitor the platelet and take circumspect application of antiplatelet drugs.

It is important to note that there are several limitations in our study. First, a larger-scale, multicenter, prospective investigation based on the relationship between thrombocytopenia and respiratory failure is needed to provide more convinced evidence. Second, the detailed molecular mechanisms underlying the process of platelet generation in lungs and how low PaO_2_ affected this process were not illuminated. In spite of these limitations, we believe that our results are the first to provide the correlation between lung diseases and thrombocytopenia with data from both clinical studies and mouse models. We anticipate that future studies will focus on identification of mechanisms underlying pulmonary thrombocytopenia through mouse models of lung injury.

## Conclusions

PIN relatively easily results in thrombocytopenia and thrombocytopenia induced by low PaO_2_ might be associated with impaired thrombopoiesis in lungs.

## Supplementary Information

**Additional file 1.** Pathogen does not affect the distribution of oxygen partial pressure in pulmonary infection patients accompanying by respiratory failure.

**Additional file 2.** Analysis on Pathogens Associated with Thrombocytopenia in Pulmonary Infection Patients accompanying by Respiratory Failure.

**Additional file 3.** Hypoxia reduce the megakaryocytes in marrow (immunofluorescence).

**Additional file 4.** Hypoxia reduce the megakaryocytes in spleen (immunofluorescence).

## Data Availability

All data generated or analyzed and material used during this study are available from the corresponding author on reasonable request.

## Abbreviations

PIN: Pulmonary Infection
UTI: Urinary Tract Infection
MKs: Megakaryocytes
RF: Respiratory Failure
COPD: Chronic Obstructive Pulmonary Disease
PaO_2_: Oxygen Partial Pressure
ICD-10: International classification of diseases code-10
UTI: Urinary tract infection
ITI: Intestinal tract infection
CPIS: Clinical pulmonary infection score
APACHE II: Acute Physiology And Chronic Health Evaluation II
PLTs: Blood platelets in angular vein
PLT_post_: Blood platelets in left ventricle
PLT_pre_: Blood platelets in right ventricle
∆PLT_post-pre_: The difference between the PLT_post_ and the PLT_pre_
