# Supplementary figures and images for: Low arterial oxygen partial pressure induces pulmonary thrombocytopenia in patients and a mouse model
Source: BMC Pulm Med. 2021 Jan 6;21:3. doi: 10.1186/s12890-020-01381-7 (PMC7789170; doi:10.1186/s12890-020-01381-7)

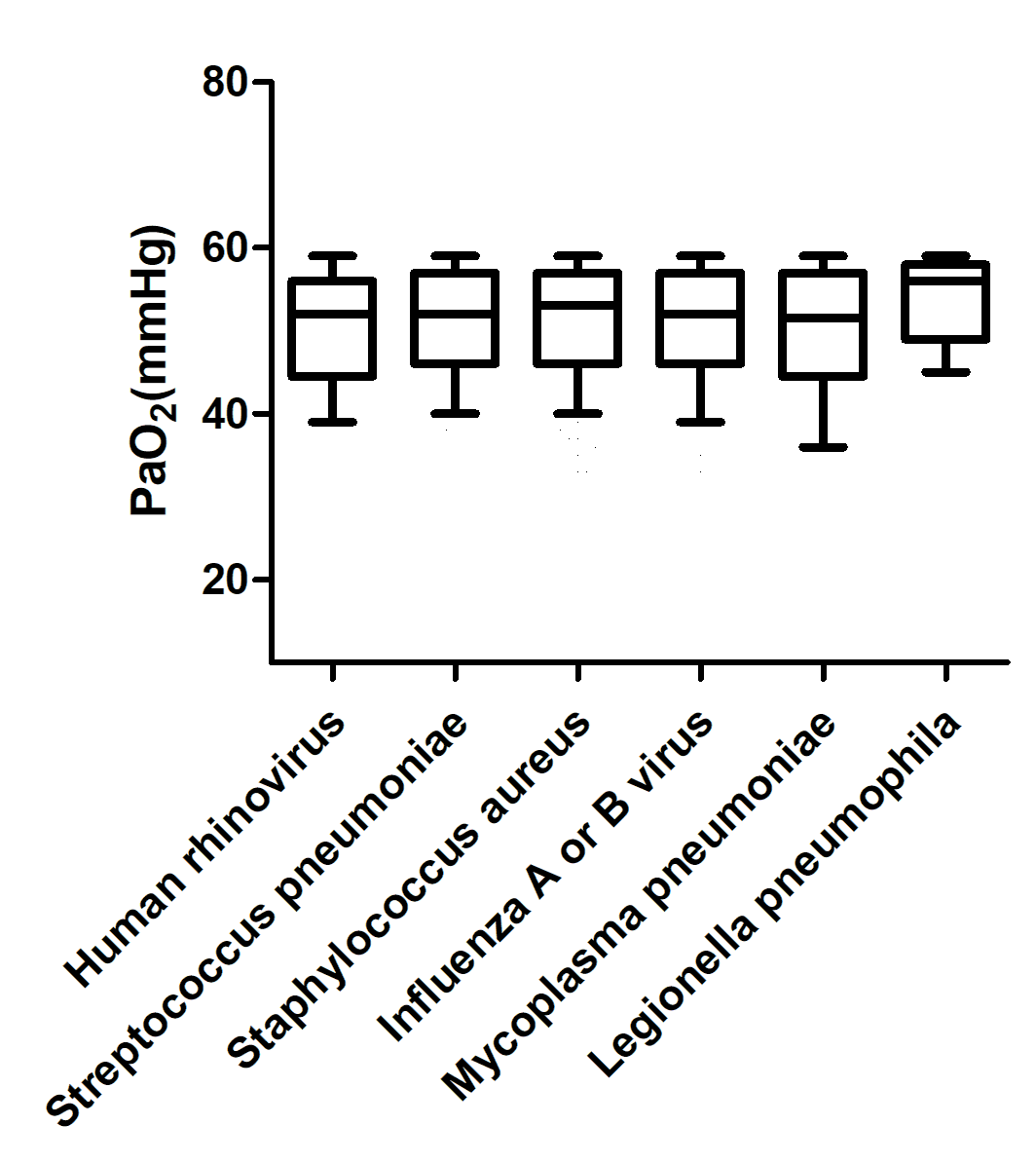

Supplement: Supplementary file 1 — Additional file 1. Pathogen does not affect the distribution of oxygen partial pressure in pulmonary infection patients accompanying by respiratory failure. [file 12890_2020_1381_MOESM1_ESM.tif]

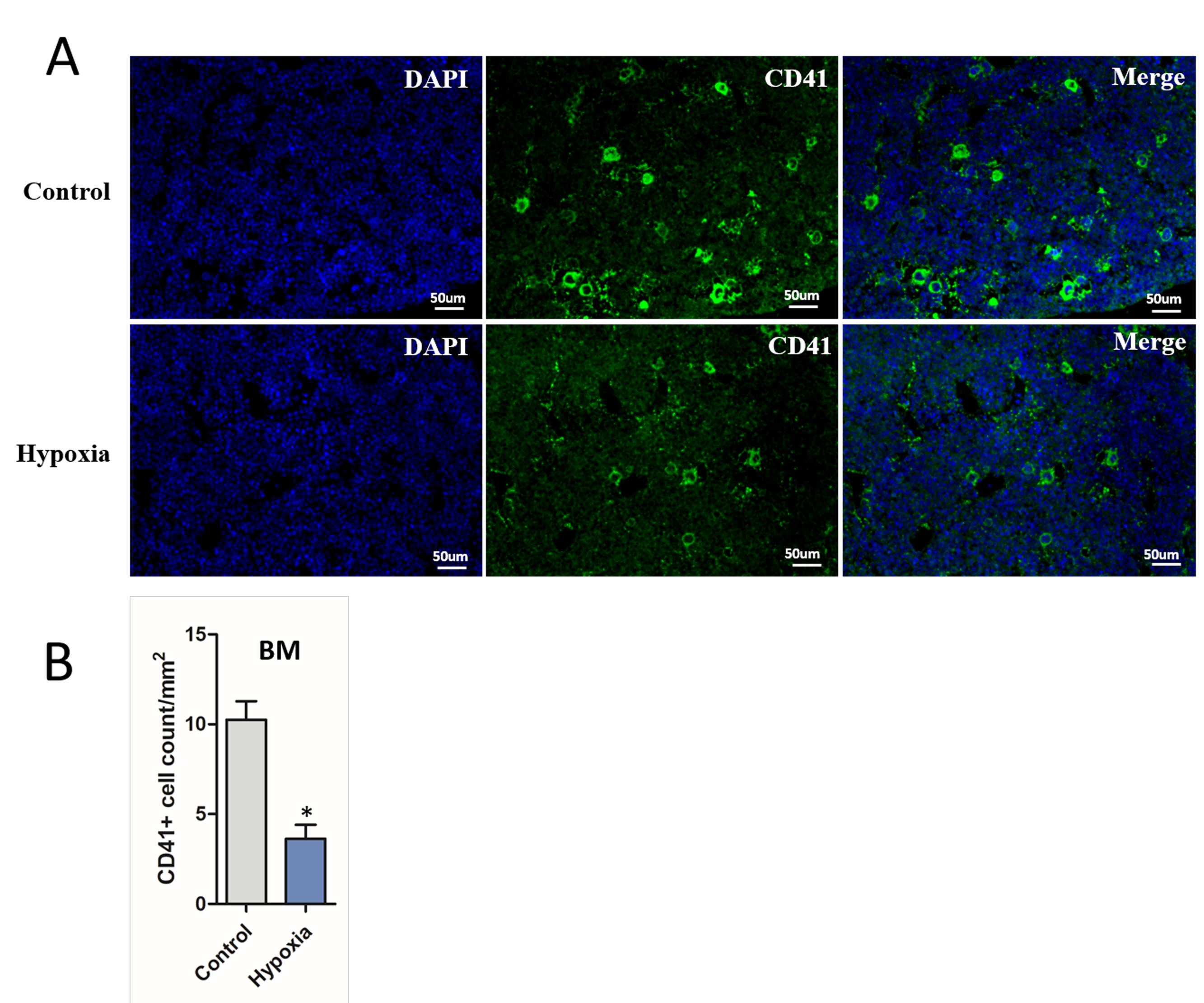

Supplement: Supplementary file 3 — Additional file 3. Hypoxia reduce the megakaryocytes in marrow (immunofluorescence). [file 12890_2020_1381_MOESM3_ESM.tif]

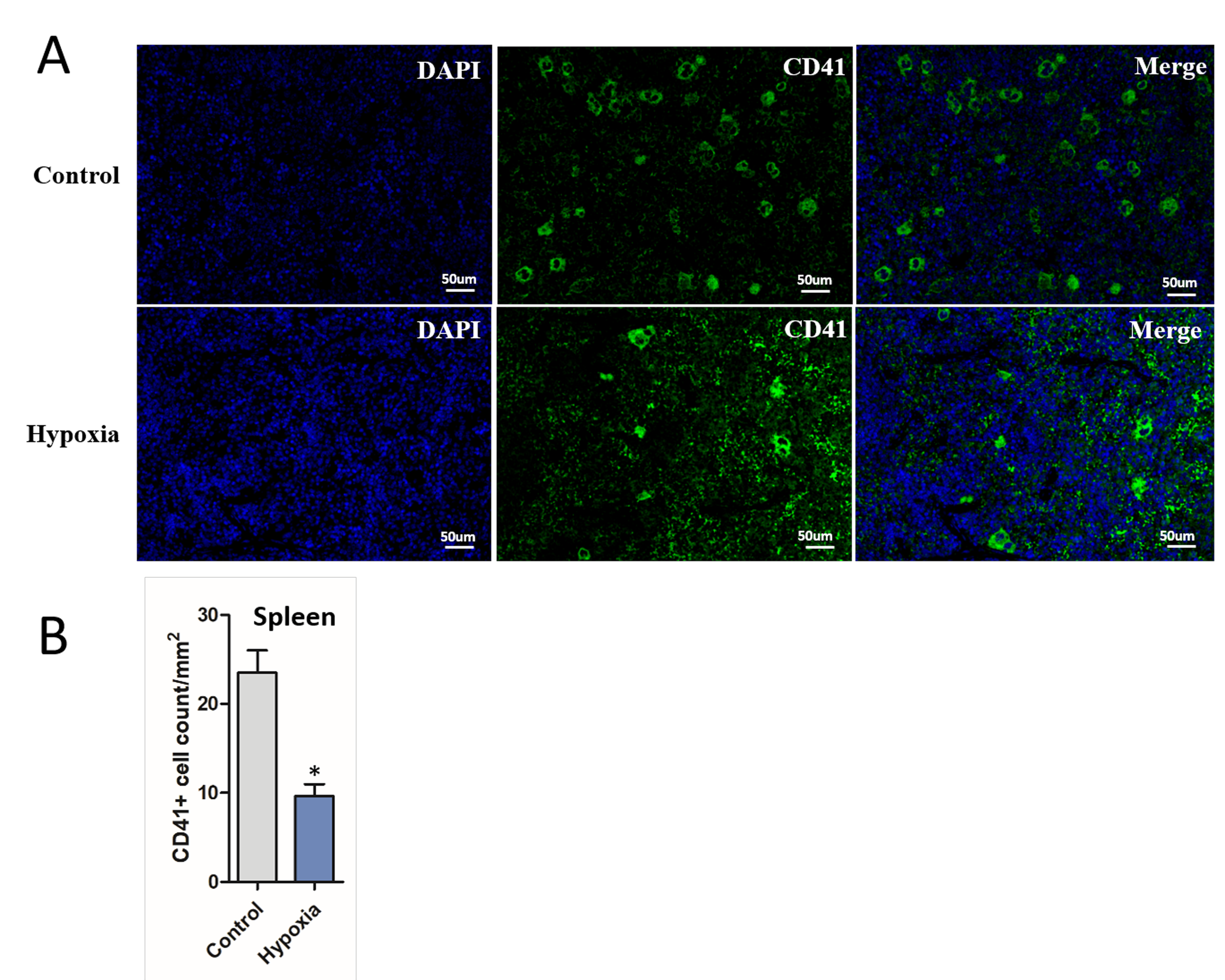

Supplement: Supplementary file 4 — Additional file 4. Hypoxia reduce the megakaryocytes in spleen (immunofluorescence). [file 12890_2020_1381_MOESM4_ESM.tif]
