# Supplementary material for: Low arterial oxygen partial pressure induces pulmonary thrombocytopenia in patients and a mouse model
Source: BMC Pulm Med. 2021 Jan 6;21:3. doi: 10.1186/s12890-020-01381-7 (PMC7789170; doi:10.1186/s12890-020-01381-7)
Supplement: Supplementary file 2 — Additional file 2. Analysis on Pathogens Associated with Thrombocytopenia in Pulmonary Infection Patients accompanying by Respiratory Failure. [file 12890_2020_1381_MOESM2_ESM.docx]

**Supplementary Table 1**

**Analysis on Pathogens Associated with Thrombocytopenia in Pulmonary Infection Patients accompanying by Respiratory Failure**

| **Variables** | **Univariate Analysis** | | | **Multivariate Analysis^#^** | | |
| --- | --- | --- | --- | --- | --- | --- |
|  | **OR** | **95%CI** | ***p* Value** | **OR** | **95%CI** | ***p* Value** |
| **Pathogen detected**^+^ |  |  |  |  |  |  |
| Human rhinovirus | 0.09 | 0.004-1.79 | 0.11 | 0.18 | 0.006-5.11 | 0.32 |
| Streptococcus pneumoniae | 0.11 | 0.007-1.81 | 0.12 | 0.24 | 0.01-5.67 | 0.38 |
| Staphylococcus aureus | 0.16 | 0.01-2.63 | 0.20 | 0.38 | 0.01-8.89 | 0.55 |
| Influenza A or B virus | 0.36 | 0.021-5.90 | 0.47 | 0.88 | 0.04-20.23 | 0.94 |
| Mycoplasma pneumoniae | 0.36 | 0.02-6.52 | 0.49 | 0.70 | 0.03-17.43 | 0.83 |
| Legionella pneumophila | 0.17 | 0.005-5.45 | 0.31 | 0.58 | 0.01-24.74 | 0.78 |

^+^Pathogen detected, 409 patients who had at least one specimen available for bacterial or viral testing.

^#^After adjusted by APACHE II scores, PaO_2_ ,COPD.

OR, odds ratio; CI, confidence interval. * Significant differences (*P*<0·05)
